# Breast cancer screening in women with extremely dense breasts recommendations of the European Society of Breast Imaging (EUSOBI)

**DOI:** 10.1007/s00330-022-08617-6

**Published:** 2022-03-08

**Authors:** Ritse M. Mann, Alexandra Athanasiou, Pascal A. T. Baltzer, Julia Camps-Herrero, Paola Clauser, Eva M. Fallenberg, Gabor Forrai, Michael H. Fuchsjäger, Thomas H. Helbich, Fleur Killburn-Toppin, Mihai Lesaru, Pietro Panizza, Federica Pediconi, Ruud M. Pijnappel, Katja Pinker, Francesco Sardanelli, Tamar Sella, Isabelle Thomassin-Naggara, Sophia Zackrisson, Fiona J. Gilbert, Christiane K. Kuhl, On behalf of the European Society of Breast Imaging (EUSOBI)

**Affiliations:** 1 Radboud University Medical Center, Geert Grooteplein Zuid 10, 6525 GA Nijmegen, Netherlands; 2 The Netherlands Cancer Institute, Plesmanlaan 121, 1066 CX Amsterdam, Netherlands; 3 Breast Imaging Department, MITERA Hospital, 6, Erithrou Stavrou Str. 151 23 Marousi, Athens, Greece; 4 Department of Biomedical Imaging and Image-guided Therapy, Division of General and Pediatric Radiology, Research Group: Molecular and Gender Imaging, Medical University of Vienna, Währinger Gürtel 18-20, 1090 Wien, Austria; 5 Hospitales Ribera Salud, Avda.Cortes Valencianas, 58, 46015 Valencia, Spain; 6 Department of Diagnostic and Interventional Radiology, School of Medicine &; Klinikum Rechts der Isar, Technical University of Munich, Munich (TUM), Ismaninger Str. 22, 81675 München, Germany; 7 Department of Radiology, Duna Medical Center, Budapest, Hungary; 8 Division of General Radiology, Department of Radiology, Medical University Graz, Auenbruggerplatz 9, 8036, Graz, Austria; 9 Department of Radiology, University of Cambridge, Cambridge Biomedical Campus, Hills road, Cambridge, CB20QQ UK; 10 Radiology and Imaging Laboratory, Carol Davila University, Bucharest, Romania; 11 Breast Imaging Unit, IRCCS Ospedale San Raffaele,, Via Olgettina 60, 20132 Milan, Italy; 12 Department of Radiological, Oncological and Pathological Sciences, Sapienza University of Rome, Viale Regina Elena, 324, 00161 Rome, Italy; 13 Department of Imaging, University Medical Centre Utrecht, Utrecht University, Heidelberglaan 100, 3584 CX Utrecht, Netherlands; 14 Dutch Expert Centre for Screening (LRCB), Wijchenseweg 101, 6538 SW Nijmegen, Netherlands; 15 Department of Radiology, Breast Imaging Service, Memorial Sloan Kettering Cancer Center, 300 E 66th Street, New York, NY 10065 USA; 16 Unit of Radiology, IRCCS Policlinico San Donato, San Donato Milanese, Milan, Italy; 17 Department of Biomedical Sciences for Health, Università degli Studi di Milano, Via Morandi 30, 20097 San Donato Milanese, Milan, Italy; 18 Department of Diagnostic Imaging, Hadassah Hebrew University Medical Center, Jerusalem, Israel; 19 Department of Radiology, Sorbonne Université, APHP, Hôpital Tenon, 4, rue de la Chine, 75020 Paris, France; 20 Diagnostic Radiology, Department of Translational Medicine, Faculty of Medicine, Lund University, Skåne University Hospital Malmö, SE-205 02 Malmö, Sweden; 21 University Hospital of Aachen, Rheinisch-Westfälische Technische Hochschule, Pauwelsstraße30, 52074 Aachen, Germany

**Keywords:** Early detection of cancer, Breast density, Mammography, Magnetic resonance imaging, Decision-making, shared

## Abstract

**Abstract:**

Breast density is an independent risk factor for the development of breast cancer and also decreases the sensitivity of mammography for screening. Consequently, women with extremely dense breasts face an increased risk of late diagnosis of breast cancer. These women are, therefore, underserved with current mammographic screening programs. The results of recent studies reporting on contrast-enhanced breast MRI as a screening method in women with extremely dense breasts provide compelling evidence that this approach can enable an important reduction in breast cancer mortality for these women and is cost-effective. Because there is now a valid option to improve breast cancer screening, the European Society of Breast Imaging (EUSOBI) recommends that women should be informed about their breast density. EUSOBI thus calls on all providers of mammography screening to share density information with the women being screened. In light of the available evidence, in women aged 50 to 70 years with extremely dense breasts, the EUSOBI now recommends offering screening breast MRI every 2 to 4 years. The EUSOBI acknowledges that it may currently not be possible to offer breast MRI immediately and everywhere and underscores that quality assurance procedures need to be established, but urges radiological societies and policymakers to act on this now. Since the wishes and values of individual women differ, in screening the principles of shared decision-making should be embraced. In particular, women should be counselled on the benefits and risks of mammography and MRI-based screening, so that they are capable of making an informed choice about their preferred screening method.

**Key Points:**

• *The recommendations in Figure 1 summarize the key points of the manuscript*

## Introduction

Breast density describes the amount of fibroglandular tissue in the breast relative to the amount of fatty tissue. The epithelial structures within the breast, i.e. the glandular lobes and the ducts, are part of this fibroglandular tissue; therefore, breast cancer mostly originates from this tissue. The amount of fibroglandular tissue is largely genetically determined and depends on hormonal stimulation. It usually decreases over time, particularly after menopause.

The fibroglandular tissue absorbs ionizing radiation (x-rays) and projects white on mammography. Consequently, it is commonly referred to as ‘dense’ tissue. Since most cancers absorb x-rays to a similar extent as fibroglandular tissue, cancers manifest as white masses on mammograms. Dense (white) tissue on mammograms can therefore hide the similarly dense (white) cancers. This means that dense tissue may prevent the detection; i.e., it can ‘mask’ cancers on mammography [1]. Only the small fraction of cancers that contain calcifications is reasonably well seen on mammography independent of the amount of dense tissue.

The distribution of the individual amount of fibroglandular tissue, and thus of mammographic densities across the female population, follows a typical bell-shaped curve (Gaussian distribution) of many biological features. In clinical practice, this biologic continuum is categorized in four large bins and is described according to the ACR BI-RADS atlas terminology as follows [2]:

- The breasts are almost entirely fatty (about 10% of the screening population [3])
- There are scattered areas of fibroglandular density (about 42% of the screening population)
- The breasts are heterogeneously dense, which may obscure small masses (about 40% of the screening population)
- The breasts are extremely dense, which lowers the sensitivity of mammography (about 8% of the screening population)

The latter two categories are commonly referred to as ‘dense’ breasts.

Although visual assessment of density is known to have a relatively high intra- and inter-reader variability, the visual estimation of density has been reported to have a somewhat higher correlation with breast cancer risk than automated assessments [4–6]. However, to minimize variability in the selection of women for supplemental or alternate screening based on breast density, automated methods may be preferable [4–6].

Besides the risk of masking breast cancer, women with extremely dense breasts in the screening age range have an increased risk of developing breast cancer, which is approximately twice as high as for the ‘average’ woman, and almost 4–6 times as high as in women with almost entirely fatty breasts [1, 7]. This is due to both the absolute higher amount of fibroglandular tissue within the breast and the breast composition [8]. Breast density is independent of other personal risk factors typically used for breast cancer risk prediction, and complementary when used in conjunction [9, 10]. Breast density is estimated to account for 26% of breast cancers in postmenopausal women [11]. Moreover, a higher breast density has also been associated with an increased breast cancer-specific mortality [12], albeit this data is not consistent among studies [13, 14].

## Current evidence on breast cancer screening in women with dense breasts

Screening is widely regarded as one of the most successful approaches to reducing breast cancer mortality in average-risk women and is recommended by the WHO [15] as well as EUSOBI [16]. Based on a meta-analysis of randomized controlled trials reporting on population screening, offering mammography screening to women aged 50–70 reduces breast cancer mortality by 20% [17]. Case-control studies in women actually screened show an even substantially higher mortality reduction of approximately 40% [18].

And yet, unfortunately, current screening strategies still fail to prevent death due to breast cancer in a substantial proportion of women: Among every 1000 women screened, in 8 disease-specific death is averted, but 11 still die from breast cancer [19]. This is due to the failure of timely detection of biologically relevant breast cancers, i.e. underdiagnosis.

Underdiagnosis is more of a problem in women with extremely dense breast tissue compared to other women. In women with largely fatty breasts, the sensitivity of mammography screening is 86 to 89%, meaning that only 11 to 14% of cancers present as interval cancer in between two screening rounds. This program sensitivity decreases to 62–68% in women with extremely dense breasts [20]. For full-field digital mammography (FFDM) similar poor figures were reported, with a program sensitivity of only 61% based on biannual screening [21]. There is currently little data on interval cancers by density for digital breast tomosynthesis (DBT), but it is unlikely that tomosynthesis is going to overcome the reduction in sensitivity caused by density. Several studies reported increased cancer detection rates by 20 to 40% also in women with (extremely) dense breasts [22, 23], mainly due to the detection of more spiculated masses and architectural distortions, but there is only limited evidence that this leads to reduced rates of interval cancers in these women. According to Conant et al, the sensitivity of DM and DBT was similarly based upon 1-year follow-up [23]. X-ray-based anatomic imaging modalities—being either screen-film mammography, FFDM or DBT—all seem to be heavily affected by breast density and thus lead to underdiagnosis of relevant cancers in these women.

Several studies have investigated supplemental ultrasound as a technique to improve the performance of population-based screening in women with extremely dense breasts [24–28]. On average, cancer detection increases by 2.3/1000 screens with ultrasound [26]. The added detection with ultrasound is also present when DBT screening is performed [27] and persists in follow-up rounds [26, 28]. Unfortunately, the number of false-positive examinations strongly increases with ultrasound. Reported positive predictive values for biopsy vary widely, but are commonly below 10% for findings only observed at ultrasound [26] and remain relatively low, even as specificity increases in follow-up rounds [28]. In a large prospective Japanese study conducted in women aged 40–49 years, it was shown that program sensitivity improved from 77 to 91% with the addition of an ultrasound examination [25]. Moreover, they showed that the frequency of interval cancer was reduced by 50%. Nevertheless, it is not clear whether these findings can be considered a valid reference for European screening programs, where screening focuses on women aged 50–69, where the incidence of breast cancer is much higher and where women tend to have larger, more heterogeneous breasts; the results do provide initial evidence to suggest that, for some women, ultrasound may be beneficial [24–29]. Within Europe, supplemental ultrasound has been structurally implemented in Austria for women with dense breasts (BI-RADS classes c and d). In the timeframe from 2014 to 2017, the program showed a sensitivity of 71% and a specificity of 99%. The breast cancer detection rate was similar to EU standards. However, currently, the added value of supplemental ultrasound regarding cancer detection is limited [30].

Accordingly, so far, these results have been insufficient for EUSOBI to recommend that average-risk women undergoing mammographic screening should be informed about their breast density [16].

This reluctance was explained by the following facts:

- We were not convinced that the benefit/risk ratio of supplemental screening for women with extremely dense breasts was positive.
- Many European countries do not offer any form of supplemental screening.
- Informing women about their density, in absence of high-level scientific evidence for screening alternatives, could increase anxiety and reduce screening participation.

However, this policy is now to change.

This change is prompted by an analysis of results of recent screening studies with contrast-enhanced breast MRI, particularly the DENSE trial and the EA1411 ECOG-ACRIN study.

The DENSE trial is a Dutch nationwide multicenter randomized trial in women with extremely dense breast tissue—as automatically assessed by a computer program (Volpara)—with a normal mammographic screening result [31].

Of the women invited for contrast-enhanced MRI, 59% agreed to participate (4783/8061) and underwent MRI screening. Supplemental MRI detected an additional 16.5 cancers /1,000 screens in the first round.

The interval cancer rate was 0.8/1,000, compared to 4.9/1,000 in women invited but not participating and 5.0/1,000 in women in the control group (*n* = 32,312). In other words, undergoing supplemental MRI screening reduces the frequency of interval cancers by 84%, thus effectively reducing underdiagnosis. The number of benign findings leading to recall was 79.8/1,000 with MRI screening. The PPV of MRI prompted biopsy was 26.3%, which we deem acceptable because it is similar to the PPV of biopsy reported for mammography.

That MRI indeed detects breast cancers earlier is also apparent from the number of cancers detected at the subsequent mammographic screening round, which was 2.0/1,000, as compared to 6.8/1,000 in the regular population of women with extremely dense breasts.

Furthermore, the next MRI screen (2 years later) yielded a supplemental detection rate of only 5.9/1,000, all of which were stage 0/1 and node-negative; providing further evidence that relevant cancers are detected predominantly earlier. Moreover, the number of benign lesions leading to recall becomes much smaller in the follow-up round (28.4/ 1,000), and therefore, the PPV remained stable (PPV = 23.5% in follow-up) [32].

The results of the DENSE trial have been modelled in a microsimulation model (MISCAN) to determine the long-term impact of offering breast MRI screening to women with extremely dense breasts [33]. This model was also used to explore other scenarios, for which the model used the measured sensitivity and specificity of mammography and MRI as observed in the DENSE trial, plus the estimated biological behavior of breast cancers, and information on the efficacy of treatment of breast cancers obtained from historical data. The results of this microsimulation model suggest that adding biennial MRI to biennial mammography—as was performed in the DENSE trial—would save 8.6 additional lives per 1,000 women invited, at a cost of 150,000 Euro per life, or 22,500 Euro per quality-adjusted life-year (QALY). While this is already deemed cost-effective, alternative strategies using MRI alone (without mammography) dominate this strategy in the model. For example, using MRI alone once every 4 years could be regarded as the most cost-effective screening strategy. This would save 7.6 additional lives per 1,000 women screened at a cost of 75,000 Euro per life or 11,500 Euro per QALY. In practice, MRI alone with a frequency of once every 2 to 3 years may be preferred to prevent non-detection of rapidly growing cancers, although a higher frequency may also lead to a somewhat higher false positive rate (see below).

As the costs of MRI screening are mostly influenced by the cost of the MR scans [33], there is strong interest in breast MRI with shorter scan protocols, generally referred to as abbreviated breast MRI. This may enable a higher throughput and therefore a lower cost per examination.

The EA1411 ECOG-ACRIN study was an international study (mainly conducted in the USA) with 48 sites in academic, community hospital, and private practice settings. It included 1,444 women with dense breasts (heterogeneously dense—category c, or extremely dense—category d), who underwent routine screening both by DBT and abbreviated MRI. Both screening methods were conducted in randomized order and read strictly independently of each other, in order not only to compare the performance of abbreviated MRI with that of DBT, but to also investigate the use of abbreviated MRI as a stand-alone screening method [34]. MRI protocols were variable, but were all shorter than 10 min. In the first screening round of the EA1141 study, overall cancer detection rate with MRI was 15.2/1,000, as compared to 6.2/1,000 for DBT. Respective sensitivity was 95.7% versus 39.1%. No interval cancers were observed. The positive predictive value for biopsy was somewhat lower for MRI (19.6% versus 31%), although not statistically significantly different. This is likely caused by the fact that a prior DBT needed to be available (i.e. this was a follow-up DBT screening examination), whereas none of the participants could have had a prior MRI (i.e. this was a first-round MRI examination). In summary, the ECOG-ACRIN EA1141 study shows that abbreviated MRI can have a similar success as the standard MRI protocol that was used within the DENSE trial. Moreover, the study provided further evidence that in women undergoing MRI for screening, the additional contribution of x-ray-based breast imaging is very limited [34].

In summary, there is cumulating evidence on the fact that women with dense breasts are underserved by screening with mammography or DBT alone. This evidence is available for both women with heterogeneously dense as well as extremely dense breasts. For the latter, there is now level I evidence available on the efficacy of MRI screening on reducing underdiagnosis and breast cancer-specific mortality, and on an improved benefit-risk balance of screening compared to regular, mammographic screening. While for women with heterogeneously dense breasts MRI may also improve cancer detection, the risk-benefit balance is currently less clear.

Consequently, EUSOBI will now recommend MRI screening in women with extremely dense breasts as specified in the “EUSOBI recommendations on screening women with dense breasts” section. This recommendation is independent of other recommendations for screening in women at increased risk due to, for example, family history or a personal history of breast cancer. The evidence is strongest for women aged 50 to 70. However, it could be considered to adopt the recommendations from the age at which screening is started when this is different.

Despite the currently available evidence, it is likely not possible to implement MRI screening for women with extremely dense breasts immediately and everywhere. Differences in the availability of equipment, staff and experience and the general willingness of policymakers to pay for screening tests vary from country to country and will affect the level to which these recommendations can and will be implemented.

When implementing MRI screening, it is *essential* to standardize the examinations, educate technologists, radiologists and other involved professionals, and monitor the quality of images acquired. Radiologists’ performance must also be monitored with a specific focus on the prevention of false-positive recalls, as these are considered a major burden to the healthy female population. The availability of MR-guided biopsy is essential for the introduction of breast MRI as a screening technique [35].

## Recommendations on how to inform women

Physicians who counsel women about their respective choices regarding breast cancer screening in general, and screening in women with extremely dense breasts in particular, must have expertise in the principles of screening in general, and in screening by imaging in particular.

Such expertise is usually not routinely available in primary healthcare providers.

Accordingly, EUSOBI urges radiologists to assume this important task and directly engage in informing women about the pros and cons of screening. Educating other healthcare providers might be another way to ensure that women receive correct and objective information. The following passages may serve as a guide for women’s education;

### How to explain the advantages associated with breast MRI screening in women with extremely dense breasts

Based on the modelled results of the DENSE trial [31, 33], it is through the following:

- A woman with extremely dense breasts who is never screened has a chance of a little over 5 % to die from breast cancer.
- Participation in screening with mammography every other year leads to early detection of cancer in about 7 % of women and reduces the likelihood to die of breast cancer to just over 4 %, i.e. reduces the risk to die of breast cancer by 20%.
- According to epidemiological modelling of DENSE trial results, participation in screening with MRI every other year leads to early cancer detection in about 10% of women and reduces the risk to die from breast cancer to a little over 3%, providing a mortality reduction by about 40 %.
- According to epidemiological modelling of DENSE trial results, when dying from breast cancer is prevented by MRI, a woman gains on average 15 years in good health, before she dies of another cause.
- For women who would have survived breast cancer also in absence of screening, the benefit is mainly that earlier detection of breast cancer might enable less aggressive treatment.

This reduced mortality, and potentially less aggressive treatment, comes at a price.

### How to explain the disadvantages associated with breast MRI screening:

In essence, screening in general, as well as screening by MRI in particular, has three relevant disadvantages. Women should also be thoroughly informed about these downsides of screening in order to be able to make informed choices.

#### First, the need to undergo the screening test

For women with extremely dense breasts, this currently implies undergoing a mammogram at least once, i.e. at the start of screening, to establish the presence of extremely dense breasts, and then contrast-enhanced breast MRI, either as a supplemental or stand-alone screening test, once every 2 to 4 years.

Hence, she should be informed about the need for an IV cannulation, the administration of an intravenous contrast agent and the nature of a 10-min MRI examination [35, 36]. While these examinations are in general well accepted, they are not perceived as pleasant. The administration of contrast agent implies that there is a very small risk for non-negligible (allergic) contrast reactions. Notably, these allergic or pseudo-allergic reactions are rare, and the vast majority of these events are mild.

Possible side effects of the contrast agent are as follows [37]:

- Occasionally (about 1 in 100): headache or nausea.
- Rare (less than 1 in 1,000): anaphylactoid or mild anaphylactic reactions leading to rash, mild drop in blood pressure, tachycardia, not requiring specific treatment.
- Extremely rare (less than 1 in 1,000,000): severe hypersensitive reactions (anaphylactoid or anaphylactic) with cardiovascular, respiratory of cutaneous manifestations, ranging from mild to severe, potentially life-threatening.

#### Second, the possibility of false-positive screening findings

Whenever screening findings are abnormal, further assessment is required to establish a final diagnosis to finally decide whether the finding represents breast cancer or not. Where this assessment confirms the presence of breast cancer, the respective screening finding is considered ‘true-positive’; when the assessment proves the presence of a benign change, but no breast cancer, the respective screening finding is considered ‘false positive’—possibly better understood when referred to as ‘false alarm’.

Women should be informed that supplemental screening tests in general, and screening with breast MRI in particular, when used over several years or even decades, will increase the chance that she will at least once experience the situation of a ‘false alarm’, i.e. receive a positive screening test which, after appropriate assessment, turns out to be a harmless finding. Of all positive (abnormal) screening findings, only about 30% are really cancerous; this value is similar for mammography and for MRI.

Women should also be informed about the fact that the ‘assessment’ to find out whether a positive screening finding corresponds to cancer or not will consist of some additional imaging studies for most, and/or of minimally-invasive needle biopsy for some women. Particularly the latter is an unpleasant and somewhat painful, yet generally well accepted, procedure [38]. Regardless, it is essential to minimize the need for additional procedures. Based on current literature, with mammographic screening approximately 1 in 7 women will ever need additional imaging or biopsy, with 2 or 4 yearly MRIs; this number may increase to approximately 1 in 4 to 5 women.

Where the assessment confirms the absence of breast cancer, in other words: In women where the screening finding was false-positive, women may have experienced (eventually unnecessary) fear of having breast cancer for a few days until the assessment results are available. Therefore, effort should be made to avoid false-positive findings altogether and to keep the time to the final diagnosis short.

No woman should ever be treated for breast cancer because of a false-positive screening finding. Only when pathologic review undoubtedly shows cancerous tissue should women receive treatment for breast cancer.

#### Third, the possibility of overdiagnosis

A number of cancers detected during screening would never have become symptomatic before the affected woman would have died of other causes. Diagnosis of such cancers is referred to as ‘overdiagnosis’. Unfortunately, overdiagnosis is not knowable at the individual level at the time of cancer detection. In practice, these women will generally be treated for their disease as currently there is no reliable method to determine whether a specific cancer is life-threatening or represents an ‘overdiagnosis’.

Based on the modelled DENSE data, about 25% of mammographically detected cancers (in 1.7% of women) and about 22% of MRI detected cancers (in 2.1% of women) may represent overdiagnosis [33]. These are mainly the low grade in situ, and some very indolent invasive breast cancers. Treatment is tailored to the specific biology of the disease in a given patient. Hence, while overdiagnosis cannot be prevented; the effect is mitigated by adapting the treatment to the aggressiveness of the detected cancer.

## Shared decision-making

Screening in general, and MRI screening in particular may be lifesaving. However, it should be realized that, although breast cancer is by far the most frequent type of cancer in women, and although it still represents the most or second-to-most important cause of cancer death in women, the vast majority of women (> 85%) will never develop breast cancer during their lifetimes.

Thus, while all women should be invited to undergo breast cancer screening, only a minority will ever be diagnosed with breast cancer, and only those women can benefit from early diagnosis. The remaining women will never develop breast cancer and in these women, undergoing screening cannot be beneficial (other than assuring a woman that she does not have breast cancer), but can only have negative side effects. From a population standpoint, the (substantial) benefit for the relatively few women who do develop breast cancer justifies the side effects of screening for the vast majority who remain cancer-free.

Still, mammographic screening is commonly criticized because of false-positive findings and overdiagnosis. Even though the benefit/risk ratio increases with MRI screening, in absolute numbers, both the number of false-positive screening tests and the number of overdiagnoses increase. *For the individual woman, the recognized side effects may be arguments to deviate from the population-based screening advice. This must be respected.*

Choosing not to attend a given screening program, or opting for a less efficient screening method, should be a choice that resides with the individual woman herself. Such a choice is a personal decision that should never be criticized, nor penalized, not even indirectly. However, to enable women to make an informed decision, they must be well informed by their radiologists (breast imagers) and should be able to place this information in the context of their preferences and values.

This is the hallmark of shared decision-making. In particular for screening, where multiple options are viable and justifiable, this participatory process is absolutely essential.

It obviously also implies that there is an obligation for the medical community to offer techniques that are proven effective; otherwise, the freedom of choice is essentially denied.

It should be noted that true application of shared decision-making clashes with current measures of the effectiveness of screening programs that assess quality—among other things—mainly by considering the overall participation rate. Although this is sound from a public healthcare perspective, it ignores the fact that individual women’s needs, priorities and values differ. What appears perfectly acceptable to one woman may be unacceptable to another. Of course, achieving or demonstrating a reduction of mortality on a population-wide level requires high participation rates. However, these concerns should not preclude or delay the recommendation of imaging tests that can effectively avoid premature death from breast cancer in individual women, even if such tests are not yet widely available.

Consequently, we should move away from evaluating the participation rate of a one-size-fits-all screening program towards more personalized screening. We should start assessing how a multifaceted screening program fits with the wishes of the women we intend to serve.

## Further considerations

This recommendation is only applicable to women with extremely dense breasts. Although women with less dense breasts, e.g. those with heterogeneously dense breasts, might also benefit from other screening approaches, the evidence in this field is currently insufficient to make strong recommendations for practice. Rather, we urge the medical community to also investigate the value of MRI screening for women with less dense breast tissue in high-quality trials.

In the future, other factors than breast density alone could be used to select women at average risk who would benefit most from MRI screening. For example, density could be combined with classic risk calculators in order to select a smaller fraction of women at higher risk for MRI screening [9, 39]. Likewise, patient selection using AI-assessment of screening mammograms could be employed as this would likely allow earlier detection of cancers in women with less dense breasts too [40]. However, these approaches are currently not validated in prospective studies, and it remains therefore uncertain whether they could achieve similar or even better results than the selection of women based upon density alone. Still, in the future, this may lead to other selection criteria for MRI screening than we currently recommend.

Due to continuous technical innovation, other imaging modalities may eventually offer practical advantages over the currently proposed contrast-enhanced breast MRI examinations, including contrast-enhanced mammography, several ultrasound-based techniques, MRI sequences without intravenous contrast administration and isotope-based imaging tests [41]. Unfortunately, most of these techniques have not (or only marginally) been tested in screening and any assumption about their efficacy is therefore premature. Still, some of these techniques could be considered in women at increased breast cancer risk with contraindications to MRI screening as they have a proven higher clinical sensitivity than mammography.

## EUSOBI recommendations on screening women with dense breasts

In women with extremely dense breast tissue at average risk, underdiagnosis of relevant breast cancers is a major challenge, even with high-quality 2D digital mammography or DBT screening. Therefore, these women are underserved by current mammographic screening programs.

**Fig. 1:**
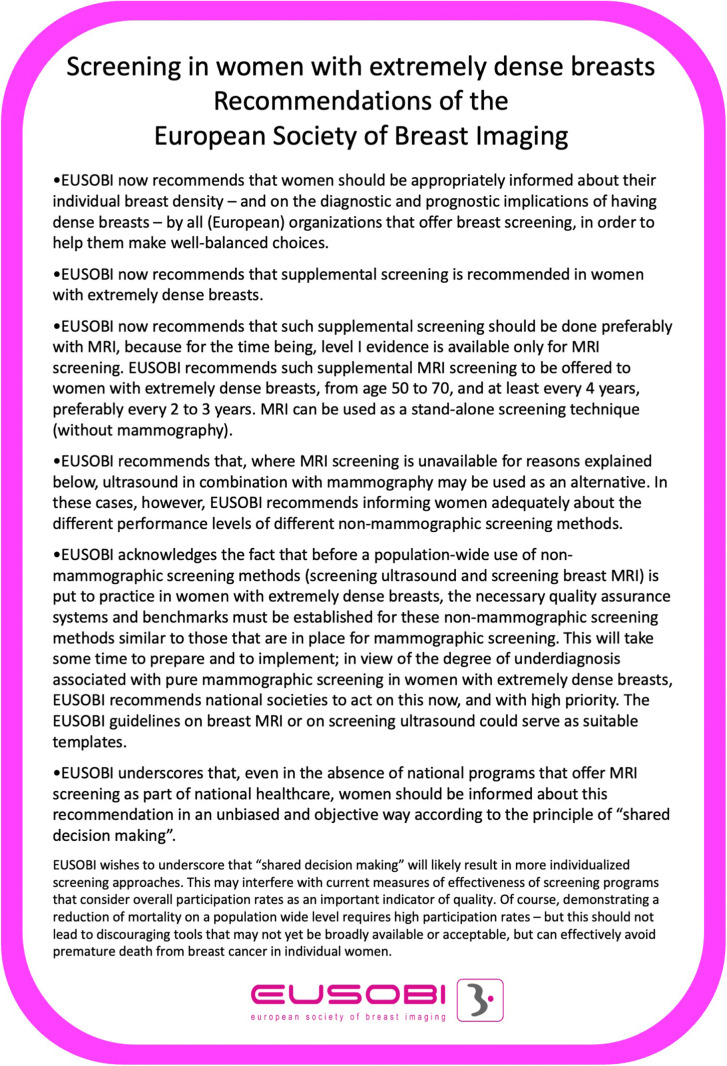
EUSOBI summary recommendations on screening women with extremely dense breasts

In view of all the above, the EUSOBI has decided to adopt the recommendation for breast cancer screening provided in Fig. 1

## Abbreviations

ACR: American College of Radiology
BI-RADS: Breast Imaging – Reporting and Data System
DBT: Digital breast tomosynthesis
DM: Digital mammography
EUSOBI: European Society of Breast Imaging
FFDM: Full-field digital mammography
MRI: (Breast) Magnetic resonance imaging
PPV: Positive predictive value
QALY: Quality-adjusted life year
WHO: World Health Organisation

## Declarations

### Guarantor

The scientific guarantor of this publication is R. M. Mann.

### Statistics and biometry

No complex statistical methods were necessary for this paper.

### Methodology

• Expert consensus based on the current literature assessment
